# Deconvolution of bulk tumors into distinct immune cell states predicts colorectal cancer recurrence

**DOI:** 10.1016/j.isci.2022.105392

**Published:** 2022-10-17

**Authors:** Donghyo Kim, Jinho Kim, Juhun Lee, Seong Kyu Han, Kwanghwan Lee, JungHo Kong, Yeon Jeong Kim, Woo Yong Lee, Seong Hyeon Yun, Hee Cheol Kim, Hye Kyung Hong, Yong Beom Cho, Donghyun Park, Sanguk Kim

**Affiliations:** 1 Department of Life Sciences, Pohang University of Science and Technology, Pohang 790-784, Korea; 2 Precision Medicine Center, Future Innovation Research Division, Seoul National University Bundang Hospital, Seongnam13620, Korea; 3 Samsung Genome Institute, Samsung Medical Center, Seoul06351, Korea; 4 Department of Surgery, Samsung Medical Center, Sungkyunkwan University School of Medicine, Seoul06351, Korea; 5 Institute for Future Medicine, Samsung Medical Center, Seoul06351, Korea; 6 Department of Health Sciences and Technology, SAIHST, Sungkyunkwan University, Seoul06351, Korea; 7 GENINUS Inc., Seoul05836, Korea; 8 Institute of Convergence Science, Yonsei University, Seoul120-749, Korea

**Keywords:** Health sciences, Health informatics, Oncology, Immunology, Bioinformatics, Biocomputational method, Systems biology, Cancer systems biology

## Abstract

Predicting colorectal cancer recurrence after tumor resection is crucial because it promotes the administration of proper subsequent treatment or management to improve the clinical outcomes of patients. Several clinical or molecular factors, including tumor stage, metastasis, and microsatellite instability status, have been used to assess the risk of recurrence, although their predictive ability is limited. Here, we predicted colorectal cancer recurrence based on cellular deconvolution of bulk tumors into two distinct immune cell states: cancer-associated (tumor-infiltrating immune cell-like) and noncancer-associated (peripheral blood mononuclear cell-like). Prediction model performed significantly better when immune cells were deconvoluted into two states rather than a single state, suggesting that the difference in cancer recurrence was better explained by distinct states of immune cells. It indicates the importance of distinguishing immune cell states using cellular deconvolution to improve the prediction of colorectal cancer recurrence.

## Highlights

• Distinct immune cell states predict colorectal cancer recurrence
• Methylation patterns of immune cells altered after tumor infiltration
• Combining immune cell states and clinical factors improves recurrence prediction
• The proportion of TIIC-like DCs is a crucial factor for the recurrence prediction

## Graphical abstract

**Figure:**
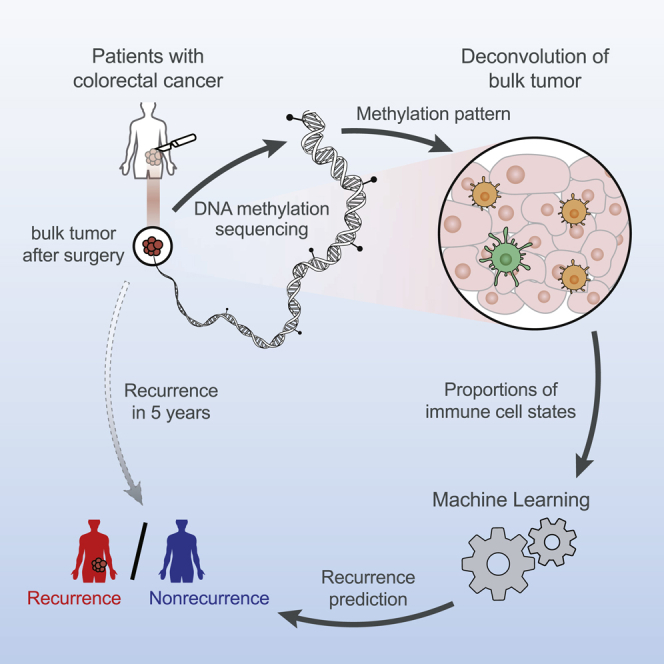

## Introduction

The prediction of recurrence in colorectal cancer patients is a challenging task. Colorectal cancer is the second leading cause of cancer-related deaths, with approximately 551,000 fatalities globally in 2018 (Bray et al., 2018). In particular, recurrence following surgery is one of the leading causes of patient mortality. After tumor excision, which is the most common treatment for colorectal cancer, 30–50% of patients experience recurrence and show a poor prognosis (Ryuk et al., 2014). For patients at a high risk of recurrence, adjuvant chemotherapy or intensive follow-up is advised to minimize the recurrence rate or detect recurrent tumors early (Desch et al., 2005; Osterman and Glimelius, 2018). Several clinical characteristics of patients are currently utilized to predict the risk of recurrence after surgery, such as tumor stage (Osterman and Glimelius, 2018), metastasis (Ryuk et al., 2014), or MSI status (Walker et al., 2014), although the prediction performance is still poor. The identification of new predictive markers for colorectal cancer recurrence is highly required in the field.

Recently, tumor-infiltrating immune cells (TIICs) have been proposed as promising prognostic markers, as their cellular and molecular mechanisms in cancer immunity have been elucidated (Fridman et al., 2012, 2017). Immune cells infiltrate into tumors and affect cancer progression and development by recognizing antigens expressed by tumor cells. Thus, the type and characteristics of immune cells in tumors have been evaluated as promising indicators for predicting the clinical outcomes of colorectal cancer patients, such as the survival rate (Galon et al., 2006). Tumor-infiltrating dendritic cells (TIDCs), for example, initiate tumor immunity by transporting tumor-associated proteins from the tumor to the lymph nodes in a CCR7-dependent manner (Gardner and Ruffell, 2016). The presence of immunogenic TIDCs in the tumor microenvironment correlated with a favorable outcome (Schwaab et al., 2001). As another example, colorectal cancer patients who have a high infiltration of CD8 effector and memory T cells in tumors have better overall survival (Pagès et al., 2009).

The relevance of TIICs in cancer prognosis has led to the development of cellular deconvolution methods, which calculate the immune cell proportions in tumors. Deconvolution methods are widely applied in cancer research (Chakravarthy et al., 2018; Craven et al., 2021) because they can be used to estimate the fraction of cell types using omics data of bulk tumors. The methods use mathematical equations to calculate the proportion of each cell type in a bulk tumor, assuming that the gene expression of bulk tumors is a weighted sum of expression profiles of various cell types. MethylCIBERSORT (Chakravarthy et al., 2018), for example, dissects each cell type’s contribution to the aggregated methylation signals in bulk tumors based on the methylation reference profiles of various cell types. Since those deconvolution methods heavily rely on the availability of accurate references, the investigation of the cell types for which reference omics data are not offered is limited.

Despite reports that the transcriptome and epigenome of immune cells might be drastically altered after tumor infiltration (Mehdi and Rabbani, 2021), omics data of immune cells from peripheral blood are still employed as reference profiles to examine the prognostic landscape of TIIC using deconvolution methods (Chakravarthy et al., 2018; Craven et al., 2021). The tumor microenvironment can influence the expression and methylation patterns of immune cells, causing them to differentiate into cancer-associated (procancer or anticancer) cell types (Mehdi and Rabbani, 2021). Upon dendritic cell maturation or CD8^+^T cell differentiation, DNA sequences containing transcription binding sites and promoters of the genes that control immune cell functions show dramatic alterations in methylation patterns, resulting in gene expression changes (Scharer et al., 2013; Zhang et al., 2014).

In this study, we examined the landscape of distinct immune cell states in bulk tumors and constructed a machine learning (ML) framework to predict colorectal cancer recurrence. To do so, we generated methylome data of various immune cell types from both tumors and peripheral blood and trained a cell deconvolution method to estimate the cellular proportion of TIIC-like and peripheral blood mononuclear cell (PBMC)-like cells in bulk tumors. We postulated that state-altered (TIIC-like, such as procancer or anticancer) and state-maintaining (PBMC-like, such as immature or bystander) immune cells coexisted in bulk tumors, each playing a different role in cancer immunity. We built an ML model to predict the recurrence of colorectal cancer patients based on the inferred proportion of TIIC- and PBMC-like cells in bulk tumors. The predictive performance of the ML model was tested using independent internal and external datasets. The ML model exhibited the best predictive performance when immune cells were deconvoluted into two different states rather than a single state, thus implying that separating the immune cell states is crucial for correctly predicting recurrence. Furthermore, our model outperforms conventional models using clinical data from patients, TNM stage, metastasis, and/or MSI status. We also examined the interpretability of the model by observing that the methylation biomarkers identified by our method were associated with the genes that control immune cell migration or activation.

## Results

### Study design

**Figure 1:**
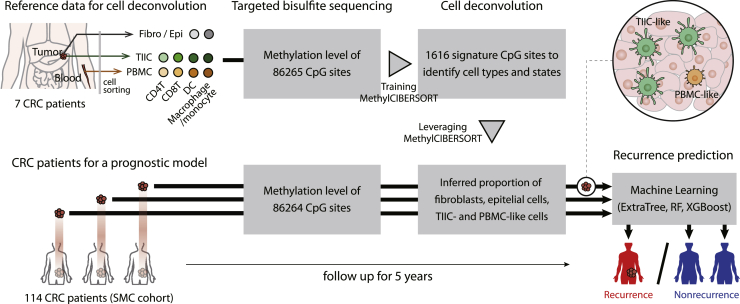
Overview of recurrence predictions based on immune cell deconvolution Recurrence in 114 colorectal cancer patients (Samsung Medical Center (SMC) cohort, 46 recurrent and 64 nonrecurrent patients) was predicted using the cell deconvolution results of patients’ bulk tumors. A deconvolution method, MethylCIBERSORT (Chakravarthy et al., 2018), selected 1,616 signature CpG sites whose methylation levels are distinct across fibroblasts, epithelial cells, tumor-infiltrating immune cells (TIICs), and peripheral blood mononuclear cells (PBMCs). TIIC and PBMC indicate CD4^+^, CD8^+^T cells, dendritic cells, and macrophages/monocytes from tumor and peripheral blood. The method trained the methylation levels of significant CpG sites to infer the proportion of cells in patients’ bulk tumors. A 5-year recurrence of patients was predicted with the inferred cellular compositions using a machine learning technique, ExtraTree (Geurts et al., 2006), Random Forest (RF) (Breiman, 2001), Extreme Gradient Boosting (XGBoost) classifiers (Chen and Guestrin, 2016).

We constructed a predictive framework that infers the composition of tumor-associated immune cells (TAICs) and PBMC-like immune cells from bulk methylome data and utilizes them to predict the risk of recurrence of patients with colorectal cancer. To identify epigenetic markers of TAICs, we compared the methylation patterns of tumor-infiltrating immune cells (TIICs) containing a relatively large number of TAICs with those of peripheral blood mononuclear cells (PBMCs) containing a relatively small number of TAICs. Specifically, we isolated epithelial cells, fibroblasts, and four types of TIICs (CD4^+^, CD8^+^T cells, DCs, and macrophages) in tumors and four types of PBMCs (CD4^+^, CD8^+^T cells, DCs, and monocytes) in peripheral blood from seven colorectal cancer patients. Then, we obtained the methylation patterns of CpG sites from the isolated cells by targeted bisulfite sequencing (Figure 1). Through a principal component analysis, we observed that the methylation patterns were distinct depending on whether they infiltrated into cancer or not, even for the same type of immune cells (Figure S1), suggesting that TIICs and PBMCs can be distinguished by epigenetic markers. Using the dataset, we (i) built a cell deconvolution model inferring TIIC- and PBMC-like immune cells in bulk tumors and (ii) developed a machine learning-based model predicting the recurrence of colorectal cancer patients by using the inferred cellular compositions. To validate the prediction performance, we used 114 colorectal cancer patients from the Samsung Medical Center (SMC) cohort, with 46 exhibiting recurrence and 64 exhibiting nonrecurrence based on an average 5-year follow-up after tumor resection.

We built a deconvolution model for the TIIC + PBMC approach and two negative control models using only methylation patterns of either TIICs (TIIC-based approach) or PBMCs (PBMC-based approach) (Figure S2). To construct deconvolution models, we leveraged MethylCIBERSORT (Chakravarthy et al., 2018), which automatically detects signature methylation patterns and created a model to infer the proportions of cell types from bulk tumor data. Using MethylCIBERSORT, we defined three methylation signatures for TIIC + PBMC-, TIIC-, and PBMC-based approaches, each of which consists of 1,616, 423, and 538 CpG sites, respectively (Table S1), and subsequently trained three deconvolution models for the three approaches. Using the trained deconvolution models, we inferred cellular compositions in bulk tumors of 114 colorectal cancer patients, which were later used to predict the recurrence of the patients.

With the inferred cellular compositions, we developed machine learning-based models to predict the recurrence of colorectal cancer patients. In antitumor immunity, interactions between various immune cell types are crucial (Luca et al., 2021; Steen et al., 2021). To train the association of immune cell proportions with cancer recurrence, we used a machine learning technique, the ExtraTree classifier (Geurts et al., 2006). We expect that there will be certain combinations of immune cells that can predict cancer recurrence. To this end, we tested all combinations of ten cell types in our machine learning pipeline. To obtain reliable accuracy estimation, we randomly split the dataset of 114 patients at a ratio of 7:3 to obtain 70% as the training set and the remaining 30% as the test set. We repeated this random splitting 100 times and obtained 100 accuracy measurements for each cell type combination. Based on the average accuracy measurement, we chose the top performing cell type combinations.

### Performance of the TIIC + PBMC approach to predict recurrence of colorectal cancer

**Figure 2:**
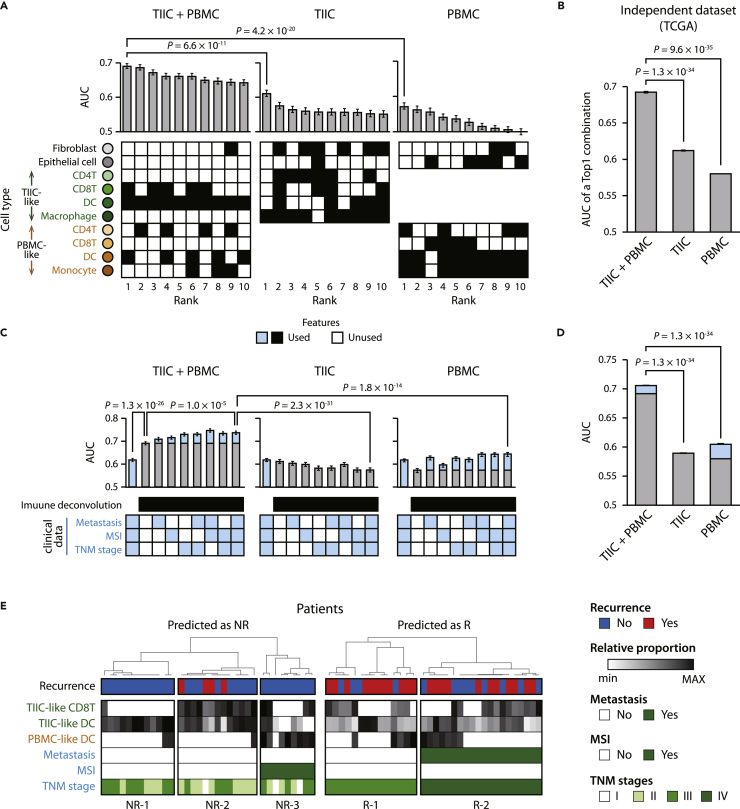
Recurrence prediction using the proportions of TIIC- and PBMC-like immune cells inferred by the bulk tumor’s methylation pattern (A) Performance comparison of predicting recurrence of 114 colorectal cancer patients based on a method of inferring the proportions of TIIC- and PBMC-like cells in a patient’s bulk tumor (TIIC + PBMC) with those of inferring the amounts of only TIIC- or PBMC-like cells (TIIC or PBMC, respectively). The AUCs of the top 10 ranked combinations of cell types are presented. Black boxes denote the combination of cell types used for prediction. Mann-Whitney U test was performed to measure significance. (B) Performance to predict recurrence of 106 colorectal cancer patients (R: 18, NR: 88) from the independent dataset TCGA. The AUCs of the top 1 ranked combination in the TIIC + PBMC, TIIC, or PBMC methods are shown. (C) Predictive performance using cell deconvolution results and clinical data (metastasis, MSI, and TNM stages). Light blue areas of bar graphs indicate the performance improvements when the clinical data were combined with cell deconvolution in predicting recurrence. Filled boxes are the features used in predictions. (D) Performances of predicting recurrence of colorectal cancer patients from TCGA using the cell deconvolution results and clinical data (MSI). (E) The recurrence annotation, the inferred proportion of cell types, and the clinical data annotation of the top 30% of patients predicted as nonrecurrence (NR) or recurrence (R). TIIC: Tumor-infiltrating immune cell; PBMC: peripheral blood mononuclear cell; TCGA: The Cancer Genome Atlas; MSI: microsatellite instability. Data are represented as mean ± SEM.

We compared the predictive performance of the three approaches, TIIC + PBMC, TIIC-based, and TBMC-based, and found that the TIIC + PBMC approach outperformed the others. The area under the curve (AUC) of the top 10 performing cell combinations of the TIIC + PBMC approach ranged from 0.67 to 0.69 (Figure 2A). In contrast, the AUCs of the top 10 cell combinations of the TIIC- and PBMC-based approaches ranged from 0.55 to 0.61 and from 0.50 to 0.57, respectively. The AUC of the top performing cell combination of the TIIC + PBMC approach was significantly higher than those of the TIIC- and PBMC-based approaches (p value = 4.5 × 10^−10^ and 8.3 × 10^−18^, respectively). Specifically, when the proportions of TIIC-like CD8^+^T cells, DCs, and PBMC-like DCs were trained, the performance to predict recurrence was the best (AUC = 0.69). DCs infiltrate the tumor to take up antigens and activate CD8^+^T cells through cross-presenting exogenous antigens to kill cancer cells (Fu and Jiang, 2018). The interaction between the two cell types is crucial for antitumor immunity. DCs and CD8^+^T cells are frequently used as good prognostic markers in cancer studies (Fridman et al., 2017). As reflected in these reports, TIIC-like DCs and CD8^+^T cells are frequently included in the top 10 cell type combinations (10 and 5, respectively).

In the TIIC + PBMC approach, we deconvoluted bulk tumors into TIIC- and PBMC-like cells because we assumed that the states of immune cells in the patients’ bulk tumors are heterogeneous, and each has a different effect on cancer recurrence. We confirmed that our assumption was valid in predicting the recurrence of colorectal cancer. We found that the proportion of TIIC-like immune cells significantly better predicts cancer recurrence when considering the methylation pattern of PBMCs together (Figure S3). Specifically, we observed that the performance using only the proportions of TIIC-like immune cells from the TIIC + PBMC approach was significantly higher than that using the proportions of TIICs from the TIIC-based approach (p value = 3.9 × 10^−7^). Similarly, the predictive performance using the proportions of PBMC-like immune cells from the TIIC + PBMC approach was higher than those from the PBMC-based approach (p value = 9.3 × 10^−3^).

We confirmed that our results are consistent when we use other tree-based machine learning techniques, random forest (RF) classifiers (Breiman, 2001) and extreme gradient boosting (XGBoost) (Chen and Guestrin, 2016). When we used the RF classifier, the AUC of the top performing cell combination in the TIIC + PBMC approach was 0.67, which was significantly higher than the AUC of the top performing cell combination in the TIIC- (AUC = 0.64 and p value = 7.3 × 10^−3^) or PBMC-based methods (AUC = 0.60 and p value = 1.5 × 10^−7^) (Figure S4). When we used the XGBoost classifier, the AUC of the top performing cell combination in the TIIC + PBMC approach was 0.67, which was significantly higher than the AUC of the top performing cell combination in the TIIC- (AUC = 0.60 and p value = 4.6 × 10^−7^) or PBMC-based methods (AUC = 0.56 and p value = 3.8 × 10^−15^) (Figure S4). This means that the successful prediction of cancer recurrence using the TIIC + PBMC deconvolution approach is robust regardless of the machine learning techniques.

We validated that the process of cell deconvolution of the patient’s bulk tumor had a significant effect on predicting colorectal cancer recurrence. When predicting cancer recurrence by training the methylation levels of the 1,616 signature methylation features from the deconvolution model, the AUC was 0.63, which was significantly lower than the AUC using the cell deconvolution result (Figure S5, p value = 1.4 × 10^−6^). Moreover, we also found that the AUC training the methylation levels of 985 signature methylation features related to TIIC-like CD8^+^T cells, DCs, and PBMC-like DCs, which were the best cell combination for predicting cancer recurrence, was still significantly lower than the AUC using the cell deconvolution result (p value = 2.1 × 10^−6^). This suggests that the inferred cellular composition, which is the cell type-level abstraction, is more useful in predicting cancer recurrence than unprocessed loci-level methylation patterns.

We further validated the predictive performance of our model using an independent dataset, The Cancer Genome Atlas (TCGA). It provides recurrence data, clinical data, and methylation data for primary tumors in 98 colon adenocarcinoma patients (TCGA-COAD) and 8 rectal adenocarcinoma patients (TCGA-READ). We found that using the proportions of fibroblasts, epithelial cells, TIIC-like macrophages, PBMC-like CD4^+^T cells, and monocytes from the TIIC + PBMC approach, cancer recurrence was predicted with an AUC of 0.69 (Figure 2B). In contrast, the AUCs of the top performing cell combinations of the TIIC- and PBMC-based approaches were 0.61 and 0.58, respectively, which were significantly lower than the AUCs of the TIIC + PBMC approach (p values are 1.3 × 10^−34^ and 1.1 × 10^−34^, respectively). Specifically, we deconvoluted the bulk tumors of TCGA patients using three deconvolution methods and predicted recurrence using a machine learning model trained with the deconvolution results of 114 colorectal cancer patients from the SMC cohort. This result suggests that our predictive strategy is also applicable to other races or cohorts, although the best feature of cell combinations was different across cohorts.

Clinical data of patients at the time of surgery, such as metastasis, microsatellite (MSI) status, and TNM stage, are associated with the recurrence of colorectal cancer after surgical excision of the primary tumor (Osterman and Glimelius, 2018; Ryuk et al., 2014; Walker et al., 2014). We found that integrating the clinical data with the cell deconvolution results improved the performance of cancer recurrence prediction (Figure 2C). Specifically, we evaluated the performance of models that integrate all combinations of clinical data with the deconvolution result of the top performing cell combination. The performance significantly increased when training the TNM stage, metastasis, and MSI status with the deconvolution result of the TIIC + PBMC approach (AUC = 0.74) compared to when only the deconvolution result was used (AUC = 0.69 and p value = 8.2 × 10^−6^). On the other hand, in the case of the TIIC- and PBMC-based approaches, even when integrated with clinical data, the AUCs were 0.57 and 0.64, respectively, confirming that the performance was significantly lower than that of the TIIC + PBMC approach integrated with clinical data (p values are 1.9 × 10^−24^ and 8.5 × 10^−14^, respectively). In the TCGA cohort, we reconfirmed that integrating the TIIC + PBMC approach with MSI status significantly improved performance (AUC = 0.70, p value = 3.0 × 10^−12^). In contrast, the AUCs of the integrating TIIC- and PBMC-based approaches with MSI status were significantly lower than the AUC of the integrating TIIC + PBMC approach with MSI status (Figure 2D, p values are 1.3 × 10^−34^ and p value = 1.3 × 10^−34^, respectively). For the cross-cohort validation with TCGA cohort, the integration of TNM stage with the deconvolution result showed poor prediction performance (Figure S6). Notably, we observed that the contribution of the deconvolution result to the -prediction performance is significant in the integrative approach with the clinical data. When only the clinical data were used, performances were significantly lower than using the deconvolution results together in both SMC and TCGA cohorts (Figures 2C and S6, SMC cohort: AUC = 0.61 and p value = 9.2 × 10^−22^; TCGA cohort: AUC = 0.50 and p value = 2.8 × 10^−39^).

The class of recurrent and nonrecurrent CRC patients for the SMC cohort was balanced (42%:58%), but that for the TCGA cohort was not (17%:83%). To evaluate the performance insensitive to the class imbalance of the TCGA cohort, we measured an additional metric, Cohen’s Kappa, which assess the agreement between two raters. Consistent with our previous results, we observed that the performance of the TIIC + PBMC approach was significantly higher than those of TIIC- and PBMC-based approaches (Figure S7, p values are 5.5 × 10^−30^ and 2.3 × 10^−35^, respectively.) Specifically, the average of Cohen’s Kappa of the TIIC + PBMC was 0.19, whereas those of TIIC- and PBMC-based approaches were 0.13 and 0.11, respectively. In addition, the improved performance was consistently observed in the results integrating clinical data (Figure S8).

Recently, tumor location and the presence of adjuvant therapy has been suggested to have prognostic impact in colorectal cancer (Shida et al., 2020). We investigated the impacts of the two clinicopathological characteristics in predicting cancer recurrence and found that they were not effective in the prediction. There was no significant difference in tumor location and treatment between recurrent and nonrecurrent patients from the SMC cohort (Figure S9). When it comes to using machine learning, tumor location and treatment showed poor performances to predict cancer recurrence (Figures S10 and S11, AUC ranged from 0.50 to 0.58). In addition, the integration of the two characteristics with the immune cell deconvolution resulted in no improvement of predictive performances. We excluded the features of tumor location and adjuvant therapy from the following analyses.

To interpret the machine learning model to predict the recurrence of patients with colorectal cancer, we compared the distributions of cell type proportions and clinical data used as prediction features for patient groups predicted as nonrecurrence or recurrence. We found that the patient groups predicted as nonrecurrence and recurrence were divided into three and two groups, respectively (Figure 2E). The first group predicted to have nonrecurrence (NR-1) showed a high frequency of TIIC-like DCs in patients’ bulk tumors. Immunogenic DCs play an important role in anticancer immunity, such as presenting tumor antigens and delivering antigens to lymph nodes for adaptive immunity against tumors (Gardner and Ruffell, 2016). This group corresponds to the report that colorectal cancer patients with a large amount of DC infiltration have better overall survival than those who do not (Schwaab et al., 2001). The second group predicted to have nonrecurrence (NR-2) had a large distribution of TIIC-like CD8^+^T cells and DCs. DCs activate CD8^+^T cells through cross-presenting exogenous antigen and kill tumor cells (Fu and Jiang, 2018). Crosstalk between CD8^+^T cells and DCs is crucial for antitumor immunity (Fu and Jiang, 2018). The patients in the third group predicted to have nonrecurrence (NR-3) had MSI, and their bulk tumors included a large proportion of PBMC-like DCs. Since tumors with MSI have a large amount of neoantigen, various immune cells, including antigen-presenting cells from peripheral blood, rapidly infiltrate into the tumor (Llosa et al., 2015), and as a result, it is associated with a favorable prognosis (Deschoolmeester et al., 2011). In contrast, the first group predicted as recurrence (R-1) had a small population of TIIC-like CD8^+^T cells and DCs, showing the opposite distribution of immune cells to the NR-2 group. The second group predicted to have recurrence (R-2) was characterized by metastasis, which is consistent with reports that patients with lymph node metastasis have a high recurrence rate after cancer resection surgery (Asano et al., 2017).

### Functional analysis of genes mapped to CpG sites used in cell deconvolution

**Figure 3:**
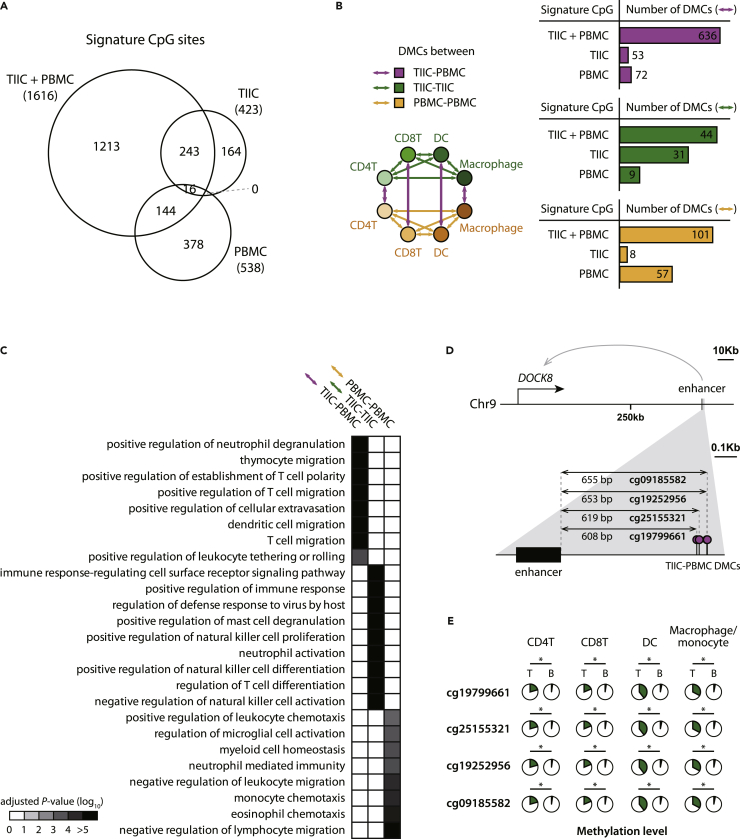
Characterization of signature CpG sites used in the integrative (TIIC + PBMC), TIIC-, and PBMC-based approaches (A) Venn diagram presenting the number of CpG sites leveraged in three approaches as signature features. (B)Number of differentially methylated CpG sites (DMCs) between TIICs and PBMCs of the same immune cell type (TIIC-PBMC) or between distinct immune cell types of TIICs (TIIC-TIIC) or PBMCs (PBMC-PBMC). (C) Functional analysis of TIIC-PBMC, TIIC-TIIC, and PBMC-PBMC DMCs in the integrative approach. To do so, we mapped CpG sites and regulatory elements and calculated the functional enrichment of their targets. Immune response-related Gene Ontology terms are only presented. The significances of the functional enrichment were corrected with Benjamini-Hochberg multiple testing correction. (D) Genomic locations of TIIC-PBMC DMCs proximal to an enhancer regulating a *DOCK8* gene. (E) Methylation levels of TIIC-PBMC DMCs in TIICs (T) and PBMCs (B) across four immune cell types. Significances of methylation level differences were corrected with Benjamini-Hochberg multiple testing correction. ∗: adjusted p value < 0.01.

We observed that the CpG sites used in the TIIC + PBMC approach tended to show significantly different methylation levels between TIICs and PBMCs of the same immune cell types. To deconvolute patients’ bulk tumors, the TIIC + PBMC-, TIIC-, and PBMC-based approaches utilized methylation patterns of 1,616, 423, and 538 CpG sites, respectively (Figure 3A). Of the 1,616 signature CpG sites used in the TIIC + PBMC approach, 636 CpG sites showed significantly different methylation levels between TIICs and PBMCs of the same immune cell types. However, in the case of TIIC- and PBMC-based approaches, only 53 and 72 sites were differentially methylated between TIIC and PBMCs, respectively (Figure 3B). These two approaches are not suitable to differentiate distinct immune states, TIIC- and PBMC-like immune cells.

We found that the DMCs between TIICs and PBMCs used in the TIIC + PBMC approach tended to be located near genes that regulate the migration of immune cells (Figure 3C). For functional characterization of TIIC-PBMC, TIIC-TIIC, and PBMC-PBMC DMCs used in the TIIC + PBMC approach, we discovered the regulatory elements (REs) located within 1,250 bp from each CpG site and mapped REs to their target genes. Using a functional enrichment test, we found that the TIIC-PBMC DMCs were significantly enriched in genes that control the process of immune cell migration to tissues, such as ‘thymocyte migration (GO:0072679)’, ‘positive regulation of cellular extravasation (GO:0002693)’, and ‘dendritic cell migration (GO:0036336)’. On the other hand, the TIIC-TIIC DMCs were significantly enriched in genes that regulate the activation of various immune cells, such as ‘positive regulation of immune response (GO:0050778)’ and ‘neutrophil activation (GO:0042119)’. The PBMC-PBMC DMCs were significantly enriched in genes important for maintaining immune cells in peripheral blood, such as ‘negative regulation of lymphocyte migration (GO:2000402)’ and ‘myeloid cell homeostasis (GO:0002262)’.

For the functional characterization by normalizing the CpG density present in base-level methylation data, functional annotation of signature CpG sites was also performed using GOmeth (Maksimovic et al., 2021; Phipson et al., 2016). We observed a similar tendency (Figure S12) to the previous results. The TIIC-PBMC DMCs are associated with genes that control immune cell migration, such as ‘positive regulation of leukocyte migration (GO:0002687)’, and genes that modulate immune cell activation, such as ‘leukocyte activation (GO:0045321)’. The TIIC-TIIC DMCs are associated with genes that regulate various immune cell activation, such as ‘myeloid leukocyte activation (GO:0002274)’. On the other hand, the PBMC-PBMC DMCs are associated with genes crucial for immune cell homeostasis, such as ‘regulation of lymphocyte chemotaxis (GO:1901623)’. These results suggest that without prior knowledge, our deconvolution model captured biologically valid information.

As an example of TIIC-PBMC DMCs, four signature CpG sites of the TIIC + PBMC approach are located around the enhancer regulating the *DOCK8* gene (Figure 3D). These CpG sites are likely to affect the expression of the *DOCK8* gene because they are close to the *DOCK8* gene and 608 bp, 619 bp, 653 bp, and 655 bp apart. The four sites were significantly hypermethylated in the TIICs of CD4^+^, CD8^+^T cells, DCs, and macrophages compared to the corresponding PBMCs (Figure 3E, p values ranged from 2.6 × 10^−2^ to 6.5 × 10^−3^). DOCK8 is a guaninenucleotide exchange factor that activates CDC42, which regulates actin polymerization and cytoskeleton rearrangement to control the migration of T cells (Xu et al., 2017) and DCs (Harada et al., 2012). *DOCK8* deficiency results in immunodeficiency and increased cancer risk, thus supporting that the epigenetic marker for *DOCK8* is associated with recurrence in colorectal cancer patients.

### Hypomethylation of the CpG sites near immunogenic DC markers in TIIC DCs

As shown in the previous results in Figure 2, we observed a lower risk of recurrence in colorectal cancer patients with a high proportion of TIIC-like DCs. We also discovered that the TIIC-like DCs is crucial for predicting cancer recurrence. Specifically, we measured feature contribution using pRF, which estimates the significance of feature importance by permutating the response variable. The TIIC-like DCs showed significant feature importance (Figure S13, p value = 9.9 × 10^−3^), which means that they are capable of increasing the performance in predicting cancer recurrence.

However, according to recent studies, not all tumor-infiltrating DCs show a favorable prognosis for colorectal cancer (Gardner and Ruffell, 2016). Tumor-infiltrating immunogenic DCs transport cancer-associated antigens to the draining lymph node and induce T cell priming to initiate anticancer immunity (Roberts et al., 2016). Thus, immunogenic DCs in the patient’s bulk tumor are associated with a favorable prognosis of patients with colorectal cancer (Schwaab et al., 2001). However, some tumor-infiltrating DCs become suppressive in the presence of cytokines such as IL-6 and M-CSF secreted by cancer cells and inhibit anticancer immunity by inactivating immune cells (Zong et al., 2016).

**Figure 4:**
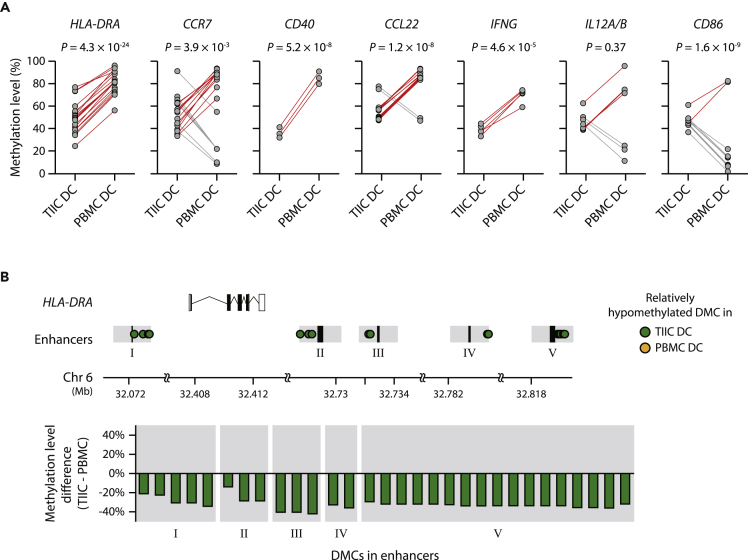
Comparison of methylation levels of regulatory elements of activated DC markers in TIIC and PBMC DCs (A) Methylation levels of the CpG sites near the noncoding regulatory elements, promoter and enhancer, controlling seven activated DC markers (*HLA-DRA*, *CCR7*, *CD40*, *CCL22*, *IFNG*, *IL12A*/*B*, and *CD86*) in TIIC and PBMC DCs. The relationships between regulatory elements and target genes identified by Marbach et al. (Marbach et al., 2016) were used. Red and gray lines indicate differentially methylated CpG sites (DMCs) relatively hypomethylated in TIIC and PBMC DCs, respectively. Paired t test was performed to measure significance. (B) Genomic locations and methylation levels of DMCs between TIIC and PBMC DCs. DMCs were discovered by using DSS (Feng et al., 2014) (p value < 0.001). Black boxes in the “Enhancers” row indicate enhancers, and gray areas represent the proximal regions within 1,250 bp from the enhancers.

To examine whether the TIIC DCs used in our study were immunogenic or suppressive, we compared the methylation levels of the CpG sites near REs regulating the gene markers of immunogenic DCs between TIIC and PBMC DCs. It is based on a report that DC development and maturation are associated with a great loss of DNA methylation of RE regulating immunogenic DC gene markers, such as *IL10* and *CCR7* (Zhang et al., 2014). We found that the sites near REs that control immunogenic DC markers tended to be significantly hypomethylated in TIIC DCs compared to PBMC DCs (Figure 4A). Specifically, we investigated the DMCs between TIIC and PBMC DCs from colon cancer patients and checked the distribution of DMCs on the sites near REs associated with seven immunogenic DC markers. The DMCs near the REs regulating five immunogenic DC gene markers (*HLA-DRA*, *CCR7*, *CD40*, *CCL22*, *IFNG*) were significantly hypomethylated in TIIC DCs (p values ranged from 3.9 × 10^−3^ to 4.3 × 10^−24^). These results showing that TIIC DCs are immunogenic support the validity of our model because patients with a high proportion of TIIC-like DCs are anticipated to have a low recurrence rate.

As an example of immunogenic DC markers, *HLA-DRA* constitutes the MHC class II (MHC-II) complex, which plays a key role in antitumor immunity by displaying neoantigen peptides and activating T cells. MHC class II molecules are frequently used as markers of immunogenic DCs (Hayashi et al., 2020). We found 30 DMCs within 2,500 bp from enhancers that regulate *HLA-DRA*, and all of them were significantly hypomethylated in TIIC DCs compared to PBMC DCs (Figure 4B). The 30 DMCs were distributed in the regions near five enhancers regulating *HLA-DRA*, and their methylation levels were 32% less on average in TIIC DCs than in PBMC DCs. These results indicated that the TIIC DCs used in this study show the properties of immunogenic DCs.

## Discussion

In this study, we predicted the recurrence of colorectal cancer using the deconvolution of bulk tumors into distinct states of immune cell types based on the methylation profiles of TIICs and PBMCs. Our results indicated that recurrence predictions were improved when using methylation profiles of TIICs and PBMCs together compared with the profiles of TIICs or PBMCs alone (Figures 2A, 2B, and S3). Our approach is based on the notion that the cellular phenotypes and functions of TIICs are heterogeneous, and they differentially affect cancer recurrence. Consistent with our results, it has been found that the prognostic capabilities of immune cell subtypes found in tumors are different. For example, in non-small-cell lung cancer (NSCLC), there were distinct amounts of subtypes of tumor-infiltrated T cells, such as naive, memory, and effector T cells (Sheng et al., 2017). Moreover, the production levels of TNF-α, an important cytokine in anticancer immunity, varied among individual subtypes of tumor-infiltrated T cells (Sheng et al., 2017). In addition to T cells, tumor-infiltrated monocytes also play multifaceted roles in anticancer immunity. They mediate both protumoral and antitumoral responses along with the tumor microenvironment (Guilliams et al., 2018; Jeong et al., 2019).

There have been efforts to predict the clinical outcomes of cancer patients by using immune cell compositions, since infiltration of immune cells into tumors is one of the important factors for clinical outcomes such as survival (Barnes and Amir, 2017), metastasis (Gonzalez et al., 2018), and drug response (Waldman et al., 2020). Therefore, many studies utilize expression or methylation profiles derived from PBMCs to infer immune cell compositions for the prediction of patients’ clinical outcomes (Chakravarthy et al., 2018; Craven et al., 2021). However, upon infiltration, the expression and methylation profiles of immune cells were significantly changed, since the tumor microenvironment affected the cells (Figures 3B, 3D, 3E, and 4). For example, the expression profiles of tumor infiltrating regulatory T cells (Tregs) were distinct from those of Tregs in PBMCs (DeSimone et al., 2016). Intriguingly, upregulated genes in tumor-infiltrating Tregs were significantly enriched for the activation of immune cells (DeSimone et al., 2016). Consistent with this finding, the methylation levels of genes related to immunogenic DCs were hypomethylated in TIIC DCs compared to PBMC DCs (Figure 4). These results suggest that methylation profiles from TIICs provide additional information about the role of immune cells in the tumor microenvironment, distinct from those in peripheral blood, thereby enabling improvement of recurrence prediction.

Our results indicated that abstraction of methylation information to depict immune cell compositions based on the deconvolution method improved the prediction of recurrence in colorectal cancer patients (Figures 2 and S6). The performance of predicting cancer recurrence was reduced when the methylation profiles derived from patients were directly used without immune cell deconvolution (Figure S5). This result suggests that the transformation of the methylation profile from locus information into cellular information using a deconvolution method enhances the predictive performance of cancer recurrence. The advantage of the information transformation before constructing a predictive model can also be found in an example of autonomous driving. Compared to directly using pixel data of camera images, known as end-to-end learning, the performance of driving path planning was improved when camera images were processed with nearby vehicles or pedestrians before planning (Ng, 2019). Camera images themselves contain too many features with unmeaningful pixels to train a model for planning a path. However, the information transformation of image pixels into objects enables a model to be trained easier and more precisely by specifying meaningful features. Likewise, because the deconvolution method transforms complicated information into simple and meaningful information, it has successfully been utilized in many cancer studies, such as for finding prognostic markers (Gentles et al., 2015) and predicting the response to chemotherapy (Ali et al., 2016) or immunotherapy (Charoentong et al., 2017).

We envision that our work here offers new perspectives for predicting colorectal cancer recurrence after surgery. First, we developed a standard model ensuring the reproducibility of predicting colorectal cancer recurrences. Briefly, we constructed a machine learning model based on cellular deconvolution of bulk tumors into two distinct immune cell states to predict cancer recurrence. The model makes robust performances in predicting cancer recurrence across independent cohorts with patients of different races (Figure 2, SMC cohort: 100% Asian, TCGA cohort: 79% White, 18% Black or African American, and 3% Asian). It suggests that recurrence prediction based on cell deconvolution across immune cell states can be applied to diverse cohorts. Second, the platform we suggested in this study makes a recurrence prediction without additional biopsy using residual tumor samples after surgery. Tumor resection is one of the most preferred treatments for colorectal cancer patients. Our platform only needs to profile the methylation patterns of a few CpG sites in the bulk tumor obtained after tumor resection. We provide a list of CpG sites to be profiled and a machine learning model for recurrence prediction through an open resource, expecting the platform facile to be employed in clinical practice. Third, deconvolution signature was synergistic to histologies which have been previously reported to have a prognostic impact on recurrence prediction. We observed that integrating the immune cell deconvolution with the clinical data improved the performance of cancer recurrence prediction (Figures 2C and 2D). The performance significantly increased when combining the immune cell deconvolution with histology currently employed in clinical practice to predict recurrence (AUC = 0.74) compared to only the histology used (AUC = 0.61 and p value = 9.2 × 10^−22^). This result suggests the applicability of cell deconvolution in clinical practice.

### Limitations of the study

A potential limitation of using immune cell states for the cancer recurrence prediction is a limited performance because of the use of a single omics layer. To improve the prediction performance, multi-omics information is required. We observed that the incorporation of clinical information such as TNM stage and metastasis with immune cell composition improved the predictive performance of recurrence in colorectal cancer patients (Figures 2C and 2D). It suggests that integration of data obtained from other layers could enhance the predictive performance of recurrence in colorectal cancer patients because recurrence of colorectal cancer is caused by heterogeneous molecular mechanisms (Augestad et al., 2017). Besides this study, other multilayered information including transcriptome (Tian et al., 2017) or genomic mutations (Hutchins et al., 2011) are used to predict the recurrence of colorectal cancer. We expect that our approach, when combined with additional information, could improve the predictive ability of recurrence in colorectal cancer patients.

## STAR★Methods

### Key resources table

**Table:** 

REAGENT or RESOURCE	SOURCE	IDENTIFIER
**Deposited data**		

Methylome from colorectal cancer patients (SMC)	This study	PRJEB50005
Processed methylation profiles from colorectal cancer patients (SMC)	This study	Zenodo (https://doi.org/10.5281/zenodo.6976028)
Methylome from colorectal cancer patients (TCGA)	TCGA Research Network	https://portal.gdc.cancer.gov/https://www.cbioportal.org/study/summary?id=coadread_tcga_pan_can_atlas_2018
Promoter-gene and enhancer-gene mappings	Marbach et al. (2016)	http://regulatorycircuits.org/

**Software and algorithms**		

Bismark	Krueger and Andrews (2011)	https://www.bioinformatics.babraham.ac.uk/projects/bismark/
Bsseq	Bioconductor	https://bioconductor.org/packages/release/bioc/html/bsseq
MethylCIBERSORT	Chakravarthy et al. (2018)	https://zenodo.org/record/1284582
CIBERSORT	Newman et al. (2015)	https://cibersort.stanford.edu/
extraTrees	CRAN	https://cran.r-project.org/web/packages/extraTrees
Xgboost	CRAN	https://cran.r-project.org/web/packages/xgboost
randomForest	CRAN	https://cran.r-project.org/web/packages/randomForest
missMethyl	1. Bioconductor	https://bioconductor.org/packages/release/bioc/html/missMethyl.html
pRF	CRAN	https://cran.r-project.org/web/packages/pRF
DSS	2. Bioconductor 3. (Feng et al., 2014)	https://bioconductor.org/packages/release/bioc/html/DSS.html
Machine-learning model predicting colorectal cancer recurrence	4. This study	Github (https://github.com/SBIlab/SGI_cancer_recurrence_methylation, https://doi.org/10.5281/zenodo.7141393)

### Resource availability

#### Lead contact

Further information and requests for resources and reagents should be directed to and will be fulfilled by the Lead Contact, Sanguk Kim (sukim@postech.ac.kr).

#### Materials availability

This study did not generate new unique reagents.

### Experimental model and subject details

#### Patient and sample collection

This study was approved by the institutional review boards of the Samsung Medical Center (approval no. SMC 2018-04-074-004). Written informed consent was obtained from all subjects. All experimental methods complied with the Helsinki Declaration. Seven colorectal cancer patients were recruited to identify signature CpG sites of TIICs and PBMCs. As a retrospective cohort to predict cancer recurrence within 5 years, 114 colorectal cancer patients were recruited. The retrospective cohort with 114 patients was designated as SMC cohort in this study. Detailed information including age, sex, and clinical records of patients in SMC cohort at the time of surgery was provided in Table S2.

#### Data curation of colorectal cancer patients (The cancer genome Atlas (TCGA) cohort)

We downloaded DNA methylation profiles of bulk primary tumors in colorectal adenocarcinoma (COAD) and rectal adenocarcinoma (READ) patients from the GDC data portal repository. We only used tumor samples from patients whose methylation profiles were measured by Illumina Human Methylation 450 (Infinium® HumanMethylation450 BeadChip). To avoid sample redundancy, meaning two or more samples from one patient, we used a sample in which methylation levels of more CpG sites were measured. Beta values were utilized as methylation levels of individual CpG sites.

Clinical data of the COAD and READ patients were downloaded from cBioPortal (https://www.cbioportal.org/, Colorectal Adenocarcinoma (TCGA, PanCancer Atlas)). The patients annotated with “0: DiseaseFree” in the “DiseaseFree Status” column were labeled as nonrecurrence, whereas the patients annotated with “1: Recurred/Progressed” were labeled as recurrence. MSI MANTIS scores higher than 0.4 were labeled microsatellite stable; otherwise, they were labeled microsatellite instable, according to a previous study (Kautto et al., 2017). The patients with annotations of TNM stage, MSI, and recurrence were analyzed for a prediction of cancer recurrence. Collectively, we curated 106 COAD and READ patients with methylation profiles and clinical information to predict the recurrence of colorectal cancer patients in the TCGA cohort. To test the effect of tumor location on recurrence prediction, we analyzed 98 COAD and READ patients having annotations of tumor locations (Colon/rectum, left/right).

### Methods details

#### Fluorescent-activated cell sorting

Tissue dissociation was performed using a Tumor Dissociation Kit (Miltenyi Biotec) according to the manufacturer’s instructions. Briefly, tissues were cut into 2–4 mm-long pieces and transferred to C tubes containing an enzyme mix. Gentle MACS programs (h_tumor_01, 02 and 03) were run in a MACSmix Tube Rotator (Miltenyi) with two 30-min incubation periods at 37°C between each run. The digested samples were filtered through a 70-μm strainer, purified using a Ficoll Paque PLUS (GE Healthcare) gradient and cryopreserved in CELLBANKER 1 (Zenoaq Resource) before fluorescent-activated cell sorting. Cell suspensions were collected by centrifugation at 200 g for 3 min, washed twice and resuspended in flow cytometry staining buffer (R&D system). Cells were stained with APC-H7–conjugated anti-CD45 at 1:200 (BD Biosciences), APC-R700-conjugated anti-CD4 at 1:200 (BD Biosciences), Alexa Fluor 647-conjugated anti-CD31 at 1:200 (BD Biosciences), PerCP-Cy5-5-conjugated anti-CD79a at 1:50 (BD Biosciences), BB515-conjugated anti-CD11C at 1:200 (BD Biosciences), BUV395-conjugated anti-CD90 at 1:200 (BD Biosciences), PE-Cy7-conjugated anti-CD68 at 1:200 (BD Biosciences), PE-CF594-conjugated anti-CD8 at 1:200 (BD Biosciences) and PE-conjugated anti-EpCAM at 1:50 (BD Biosciences) antibody for 20 min at room temperature. Tumor cells were identified by EpCAM+/CD45-; CD4^+^T cells were identified by CD4+/Thy-1+/CD45+; CD8^+^T cells were identified by CD8α+/Thy-1+/CD45+; B lymphocytes were identified by CD79A//CD45+; macrophages were identified by CD68+/CD45+; dendritic cells were identified by CD11C+/CD45+; fibroblasts were identified by Thy-1+/CD45-; and endothelial cells were identified by CD31^+^. Fluorescence-activated cell sorting was performed using a BD FACS Aria III SORP cell sorter (BD Biosciences).

#### Methylation profiling of bulk tumors and sorted cell types from colorectal cancer patients

To construct libraries for DNA methylation sequencing, 300 ng of genomic DNA was sonicated with a Covaris S220 sonicator (Covaris, Woburn, MA, USA), which generated products of 150–200 bp. Using the KAPA HyperPrep Kit (Roche, Indianapolis, IN, USA), the fragmented DNA was end-repaired, A-tailed and ligated with methylated adapters with a sample index to create a precapture DNA library. Up to 8 libraries were pooled and subjected to capture-based target enrichment using RNA baits of SureSelect Human methyl-seq (Agilent Technologies, Santa Clara, CA, USA). Hybridization was performed at 65°C for 16 h. Hybridized products were purified with streptavidin beads and then subjected to bisulfite treatment (64°C for 2.5 h) using the Zymo EZ DNA Gold kit (Zymo Research, Irvine, CA). After clean up, the bisulfite-treated libraries were PCR-amplified for 15 cycles with SureSelect Methyl-Seq PCR master mix (Agilent Technologies). Based on DNA concentration and average fragment size, libraries were normalized to an equal concentration, denatured using 0.2 N NaOH and diluted to 20 pM using hybridization buffer purchased from Illumina. Cluster amplification of denatured templates was performed according to the manufacturer’s protocol (Illumina, San Diego, CA, USA). Flow cells were sequenced using HiSeq 2500 v3 Sequencing-by-Synthesis Kits (2 × 100 bp reads).

#### Preprocessing of methylation data from colorectal cancer patients

A Bismark pipeline (Krueger and Andrews, 2011) was used to align the reads and calculate read counts of methylated or demethylated regions. We aligned sequence reads to the hg19 reference genome and retrieved genome-wide cytosine methylation reports. Smoothing bisulfite sequencing data were processed using the BSmooth function of the bsseq R package, and the CpG sites with a depth of less than 10 were filtered out. The beta values were calculated using the getMeth function of the bsseq R package to quantify the methylation level of each CpG site from the read counts.

#### Construction of DNA methylation-based cell deconvolution models

To calculate the proportions of cell types in bulk tumors from individual patients using DNA methylation profiles, we used MethylCIBERSORT (Chakravarthy et al., 2018), which is a DNA methylation-based cell deconvolution model. MethylCIBERSORT utilizes nu–support vector regression (ν-SVR) with a linear kernel based on methylation profiles of reference cell types to infer cellular proportions from the DNA methylation profiles of bulk tumors.

MethylCIBERSORT requires a “mixture” matrix, which consists of DNA methylation levels of the sites in bulk tumors from patients (CpG sites in rows and patients in columns, in this study), and a “signature” matrix, which describes the DNA methylation level of each CpG site in cell types (CpG sites in rows and cell types in columns). To construct the “signature” matrix, we first utilized methylation levels of sorted cells (TIICs: CD4^+^T cells, CD8^+^T cells, dendritic cells, macrophages, epithelial cells, and fibroblasts; PBMCs: CD4^+^T cells, CD8^+^T cells, dendritic cells, and monocytes) from 7 colorectal cancer patients, which was an independent group of patients for recurrence prediction using machine learning models. Then, we selected the CpG sites simultaneously investigated in the sorted cells and the bulk tumors. Finally, to extract signature CpG sites from sorted cells, we used FeatureSelect. V4 function provided by MethylCIBERSORT, which identifies signature CpG sites of individual cell types, based on the identification of differentially methylated CpG sites among cell types. To construct the “mixture” matrix, we extracted the methylation levels of the CpG sites of the bulk tumors from patients corresponding to the CpG sites in the “signature” matrix. Using the two matrices, we ran CIBERSORT (Newman et al., 2015) according to the manual of MethylCIBERSORT to obtain inferred cellular proportions of each patient.

#### Machine learning-based prediction of cancer recurrence using cell proportions inferred from MethylCIBERSORT

We used extremely randomized trees (Geurts et al., 2006), extreme gradient boosting (Chen and Guestrin, 2016), and random forest (Breiman, 2001), which are decision tree-based ensemble machine learning models, for the prediction of recurrence in colorectal cancer patients. To predict patient recurrence, the cellular proportions of bulk tumors from individual patients were used as an input for machine learning models. To incorporate clinical data with cellular proportions to predict patient recurrence, we used TNM stage, metastasis, and microsatellite instability (MSI), tumor locations (colon/rectum, left/right) as inputs for machine learning models. To test the effect of adjuvant chemotherapy on recurrence prediction, we used the clinical data whether the patients were treated or not for SMC cohort as inputs for machine learning models. Ascending colon, hepatic flexure colon, transverse colon, and cecum were assigned as right-sided. Rectum, sigmoid colon, splenic flexure colon, st, rectosigmoid junction rectum, and descending colon were assigned as left-sided. To validate the performance in predicting the recurrence of the SMC cohort, we used Monte Carlo cross-validation. We randomly selected 70% of the SMC cohort to train the models, and the prediction performances of the models were investigated using the remaining 30% of patients. The model constructions and performance measurements were performed 100 times iteratively (100 times Monte Carlo cross-validation).

To validate the prediction performance of the model for the TCGA cohort, we trained the model using the immune cell proportions (or incorporating clinical information) of the SMC cohort and predicted the recurrence of TCGA patients. The model constructions and performance measurements were performed 100 times iteratively. The performances of machine learning models were measured by the area under the receiver operating characteristic curve (AUC). For TCGA cohort, Cohen’s kappa was additionally used to measure performance of machine learning models because of the imbalance between recurrence and nonrecurrence. The prediction procedures were implemented in R by using the extraTrees package for extremely randomized trees, xgboost package for extreme gradient boosting, randomForest package for random forest, and ROCR package for AUC measurement, psych package for Cohen’s kappa measurement.

#### Functional characterization of the signature CpG sites used in the deconvolution model

For functional characterization of the signature CpG sites used in the deconvolution models, we mapped each CpG site into a gene whose expression is likely affected by the methylation of the CpG site. To do so, we discovered regulatory elements (REs) upstream and downstream of 1,250 bp from each CpG site based on the report that methylation levels near REs can affect the expression of their target genes (Xiong et al., 2018). We downloaded promoter-gene and enhancer-gene mappings annotated in Marbach et al. (2016) from http://regulatorycircuits.org/. Marbach et al. provided the regions of REs and their target genes defined by cap analysis of gene expression (CAGE) data from the FANTOM5 project (Forrest et al., 2014) for approximately 1,000 human tissue and cell lines, which enabled the mapping of transcription start sites with high sensitivity. Then, we analyzed GO term enrichment for the genes mapped by the CpG sites. We used immune response-related GO terms obtained from all child terms of ‘immune response (GO:0006955)’. Enrichment tests were performed to calculate an adjusted p value using the hypergeometric test and Benjamini–Hochberg correction procedure.

For the functional characterization by normalizing the CpG density, we calculated GO term enrichment of DMCs using GOmeth function from the missMethyl R package (Maksimovic et al., 2021; Phipson et al., 2016). TIIC-PBMC, TIIC-TIIC, and PBMC-PBMC DMCs were used as input of GOmeth function, and all CpG sites in the panel were used as background. We used the option, plot.bias=TRUE, and default settings were used for any other options.

#### Measurement of feature importance using pRF

To evaluate the feature importance of each cell type proportion for patient recurrence prediction, we adopted pRF (https://cran.r-project.org/web/packages/pRF), which estimated statistical significance of feature importance by permuting the response variable. The cellular proportions deconvoluted by TIIC + PBMC were used as an input of pRF, and hyperparameters, n.perms = 100, mtry = 10, and type = “classification” were used.

#### Comparison of the methylation levels of CpG sites near immunogenic DC markers between TIIC and PBMC DCs

To compare the methylation levels of TIIC and PBMC DCs, we used dispersion shrinkage for sequencing (DSS) (Feng et al., 2014), which is a statistical method to detect differentially methylated regions based on a beta-binomial regression model. Differentially methylated CpG sites (DMCs) between TIIC and PBMC DCs were identified using DSS v.2.38.0 (smoothing = TRUE and p value < 0.001). We analyzed the distributions of DMCs located 1,250 bp upstream and downstream from the REs regulating immunogenic DC marker genes. We used seven well-established DC activation/maturation marker genes, *HLA-DRA*, *CCR7*, *CD40*, *CCL22*, *IFNG*, *IL12*, and *CD86*. For *IL12*, we used REs of *IL12A* and *IL12B*, since IL12 is a complex of them. We compared the methylation levels of DMCs obtained by DSS between TIIC and PBMC DCs. The significant differences in methylation levels were quantified by paired, two-tailed and two-sample Student’s *t* tests.

### Quantification and statistical analysis

Statistical analyses of predictive performance were conducted by Mann-Whitney U test using scipy.stats modules of python. Functional enrichment tests in Figure 3 were performed using the hypergeometric test and Benjamini–Hochberg multiple testing correction. To compare methylation levels of TIIC-PBMC DMCs in TIICs and PBMCs across 4 immune cell types (Figure 3E), Student’s *t* test was used and adjusted by Benjamini-Hochberg correction (∗: adjusted pvalue < 0.01). To compare methylation levels of regulatory regions near activated DC marker genes (Figure 4A), paired t test was used.

## Data Availability

Raw sequence data derived from human samples using bisulfite sequencing have been deposited at European Nucleotide Archive (ENA) and publicly available as of the date of publication. The accession number is listed in the key resources table. Processed data matrix has been deposited at Zenodo and is publicly available as of the date of publication. The DOI is listed in the key resources table. All original code has been deposited at GitHub and is publicly available as of the date of publication. The DOI is listed in the key resources table. Any additional information required to reanalyze the data reported in this paper is available from the lead contacton request.

## Supplemental information

Document S1. Figures S1–S13

Table S1. List of methylation signatures for TIIC+PBMC-, TIIC-, and PBMC-based approaches using MethylCIBERSORT

Table S2. Clinical data of the SMC cohort at the time of surgery
