# Supplementary material for: Deconvolution of bulk tumors into distinct immune cell states predicts colorectal cancer recurrence
Source: iScience. 2022 Oct 17;25(11):105392. doi: 10.1016/j.isci.2022.105392 (PMC9636036; doi:10.1016/j.isci.2022.105392)
Supplement: Document S1. Figures S1–S13 [file mmc1.pdf]

iScience, Volume *25*

Supplemental information

Deconvolution of bulk tumors into distinct immune cell states predicts colorectal cancer recurrence

Donghyo Kim, Jinho Kim, Juhun Lee, Seong Kyu Han, Kwanghwan Lee, JungHo Kong, Yeon Jeong Kim, Woo Yong Lee, Seong Hyeon Yun, Hee Cheol Kim, Hye Kyung Hong, Yong Beom Cho, Donghyun Park, and Sanguk Kim

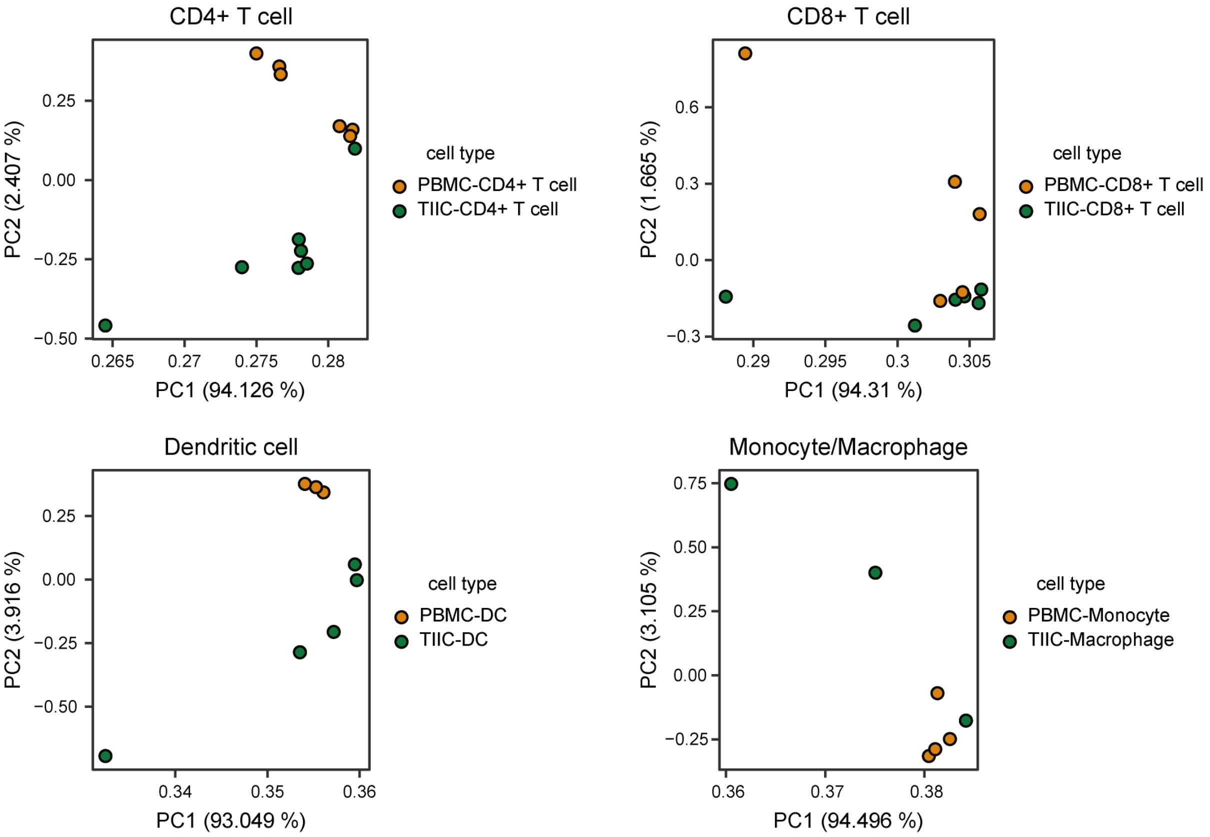

Figure S1. Principal component analysis of methylation patterns across four kinds of cell types according to their origins. Methylation levels of all CpG sites from four kinds of cell types were projected onto the first two principal components. Greenish and yellowish dots indicate tumor-infiltrating immune cells (TIICs) and peripheral blood mononuclear cells (PBMCs), respectively. Related to **Figure 1**.

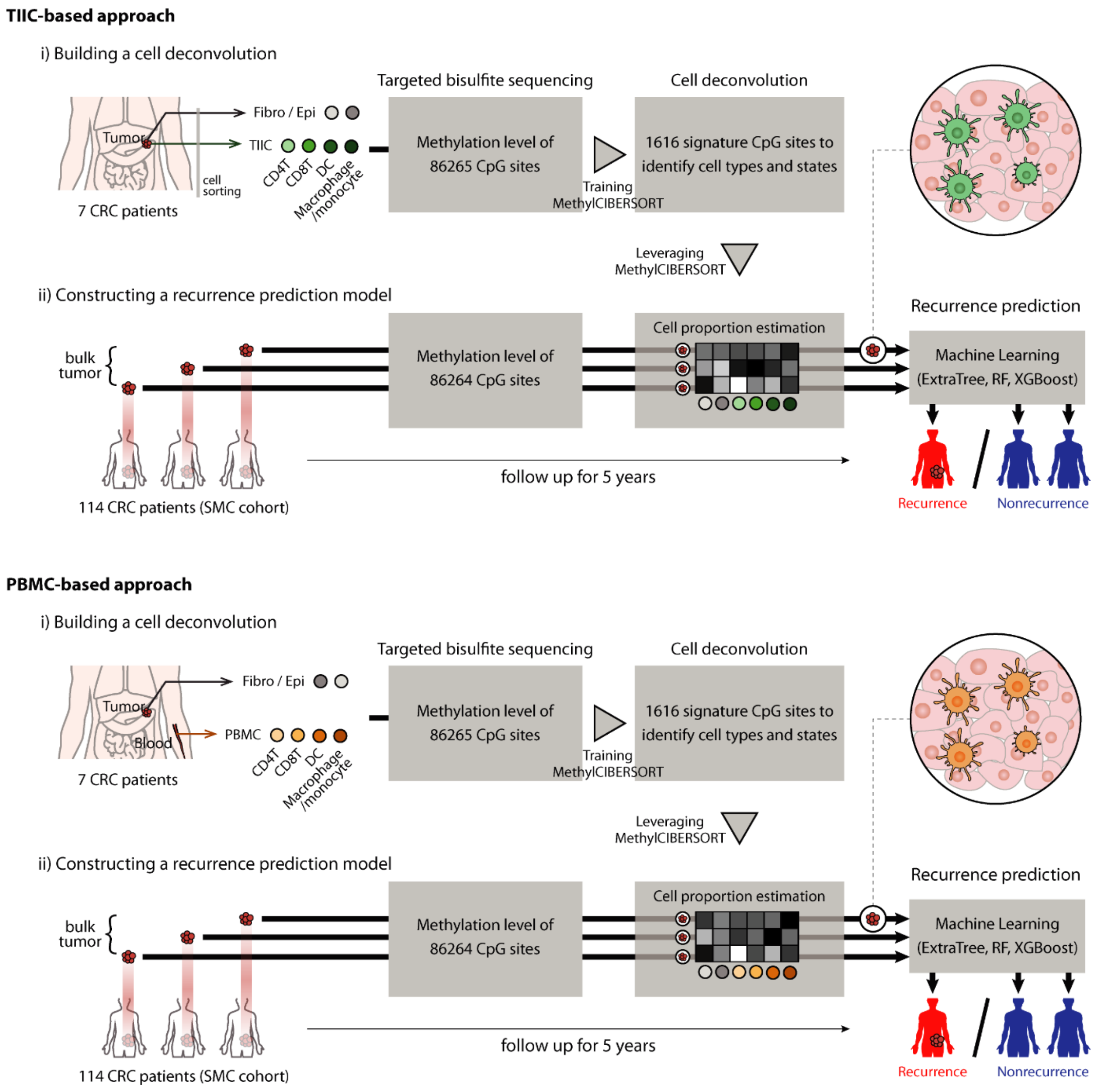

Figure S2. Overview of recurrence prediction based on TIIC- (top) and PBMC-based approaches (bottom). Recurrence in 114 colorectal cancer patients was predicted using cell-type proportions inferred by a deconvolution method. The deconvolution method infers the proportions of fibroblast, epithelial and immune cells based on the methylation patterns of corresponding sorted cell types. TIIC- and PBMC-based approaches use methylation of immune cells from the tumor and peripheral blood, respectively. Related to **Figure 1**.

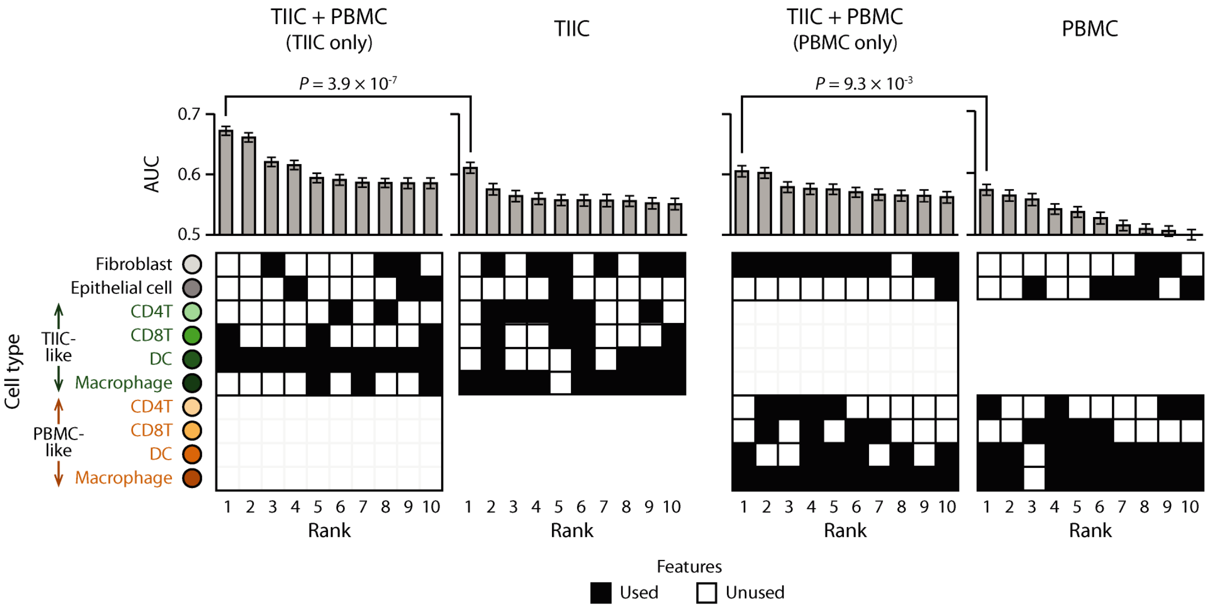

Figure S3. Performance comparison of the prediction using only the proportions of TIIC- or PBMC-like cells in the integrative approach (TIIC+PBMC) with the prediction using TIIC- or PBMC-based approaches. The AUCs of the top 10 ranked combinations of cell types are presented. Black boxes denote the combination of cell types used for prediction. Mann-Whitney U test was performed to measure significance. Data are represented as mean ± SEM. Related to **Figure 2**.

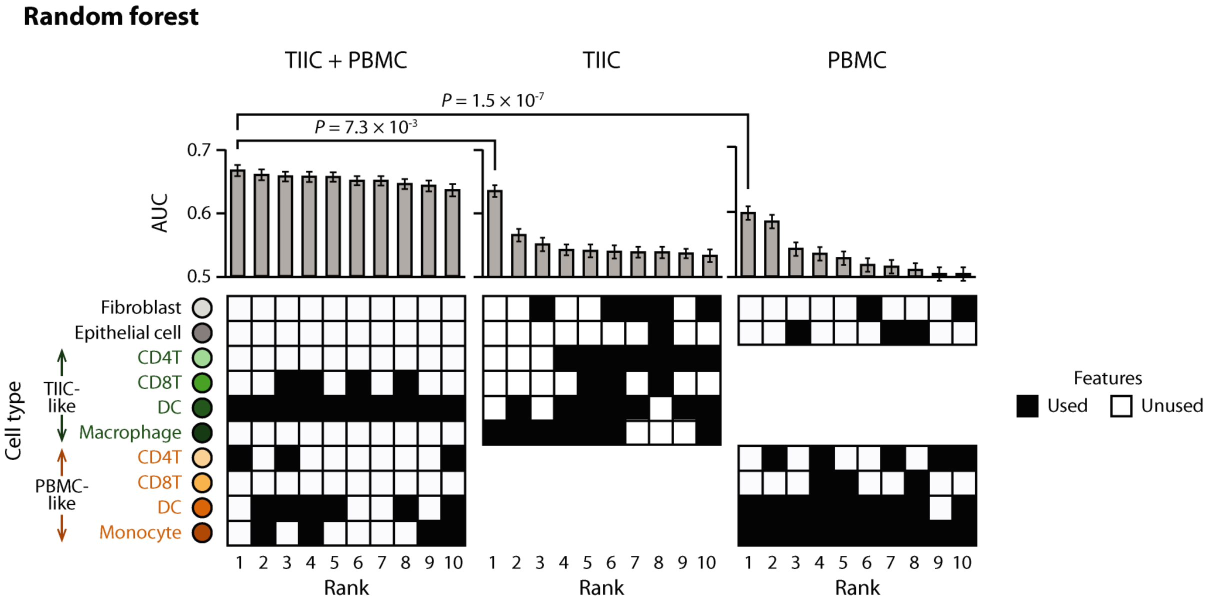

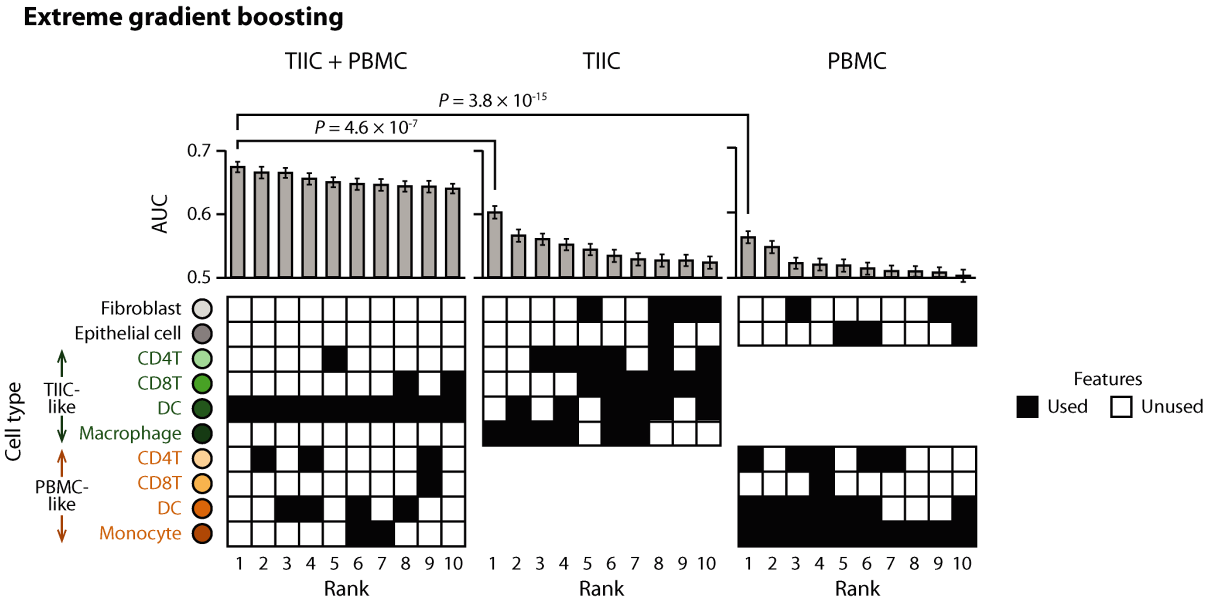

Figure S4. Prediction performances of the integrative (TIIC+PBMC), TIIC-, and PBMC-based methods trained by the random forest and extreme gradient boosting classifiers. The AUCs of the top 10 ranked cell type combinations are presented. Black boxes denote the combination of cell types used for prediction. Mann-Whitney U test was performed to measure significance. Data are represented as mean ± SEM. Related to **Figure 2**.

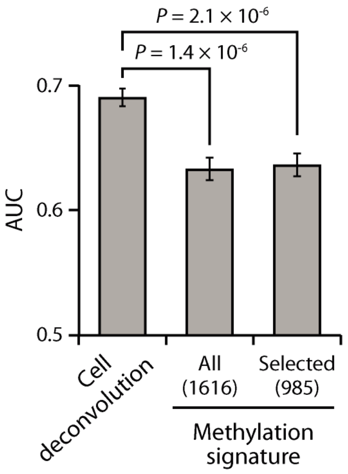

Figure S5. Performance of cell deconvolution and methylation signature to predict the recurrence of colorectal cancer patients. Left: AUC of using the cell deconvolution results (TIIC-CD8+ T cells, TIIC-DCs, and PBMC-DCs). Middle: AUC of using the methylation level of 1616 signature features used in cell deconvolution. Right: AUC of using the methylation level of 985 selected signature features specific to TIIC-CD8+ T cells, TIIC-DCs, and PBMC-DCs. Mann-Whitney U test was performed to measure significance. Data are represented as mean ± SEM. Related to **Figure 2**.

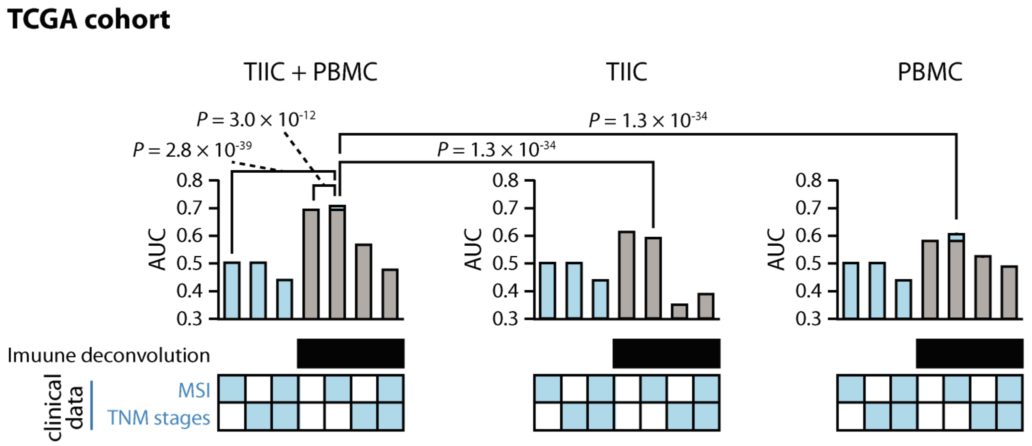

Figure S6. Prediction performance integrating cell deconvolution results and clinical data in the TCGA cohort. The cell deconvolution results of the top 1 ranked combinations in the TIIC+PBMC, TIIC, or PBMC methods were used for integration. Black and light blue boxes denote the combination of cell types and clinical data used for prediction, respectively. Mann-Whitney U test was performed to measure significance. Data are represented as mean ± SEM. Related to **Figure 2**.

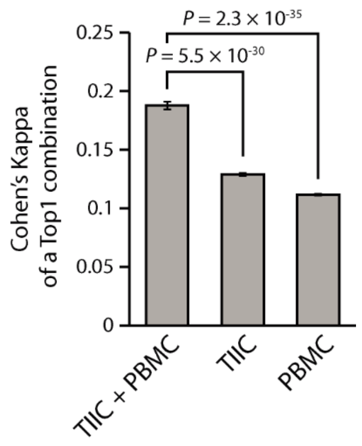

Figure S7. Performances of the TIIC+PBMC, TIIC-, and PBMC-based methods to predict cancer recurrence in the TCGA cohort using a class balance insensitive metric. The Cohen's Kappas of the top 1 ranked combinations in the TIIC+PBMC, TIIC, or PBMC methods are shown. Mann-Whitney U test was performed to measure significance. Data are represented as mean ± SEM. Related to **Figure 2**.

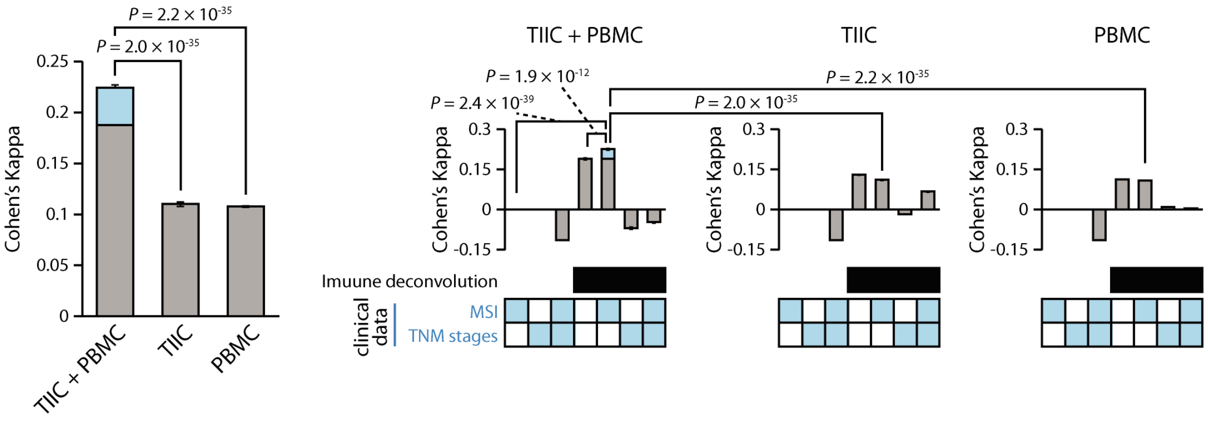

Figure S8. Prediction performance integrating cell deconvolution results and clinical data in the TCGA using a class balance insensitive metric. The cell deconvolution results of the top 1 ranked combinations in the TIIC+PBMC, TIIC, or PBMC methods were used for integration. Black and light blue boxes denote the combination of cell types and clinical data used for prediction, respectively. Mann-Whitney U test was performed to measure significance. Data are represented as mean ± SEM. Related to **Figure 2**.

		Recurrent	Nonrecurrent	P value
Adjuvant treatment	Yes	34	44	0.31
	No	12	24	
Tumor location	Colon	28	40	0.85
	Rectum	18	28	
	Left	30	46	0.84
	Right	16	22	

Figure S9. Adjuvant treatment and tumor location information of recurrent and nonrecurrent patients from the SMC cohort. The number of patients who received (Yes) and did not receive (No) adjuvant treatment at the time of surgery were presented. Ascending colon, hepatic flexure colon, transverse colon, and cecum were assigned as right-sided. Rectum, sigmoid colon, splenic flexure colon, st, rectosigmoid junction rectum, and descending colon were assigned as left-sided. P value was measured using the Fisher's exact test. Related to **Figure 2**.

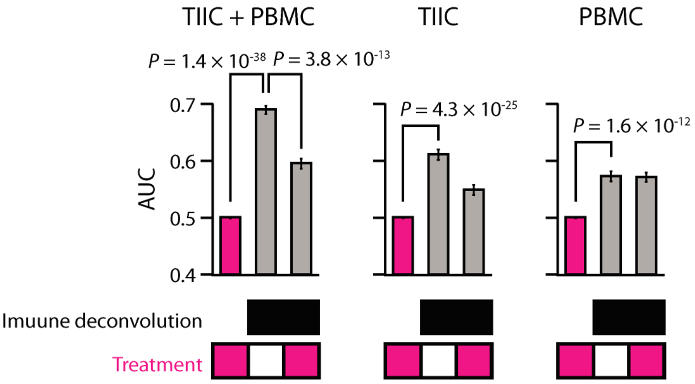

Figure S10. Performances of predicting recurrence of colorectal cancer patients from the SMC cohort using the immune cell deconvolution and adjuvant treatments. Filled boxes are the features used in predictions. Mann-Whitney U test was performed to measure significance. Data are represented as mean ± SEM. Related to **Figure 2**.

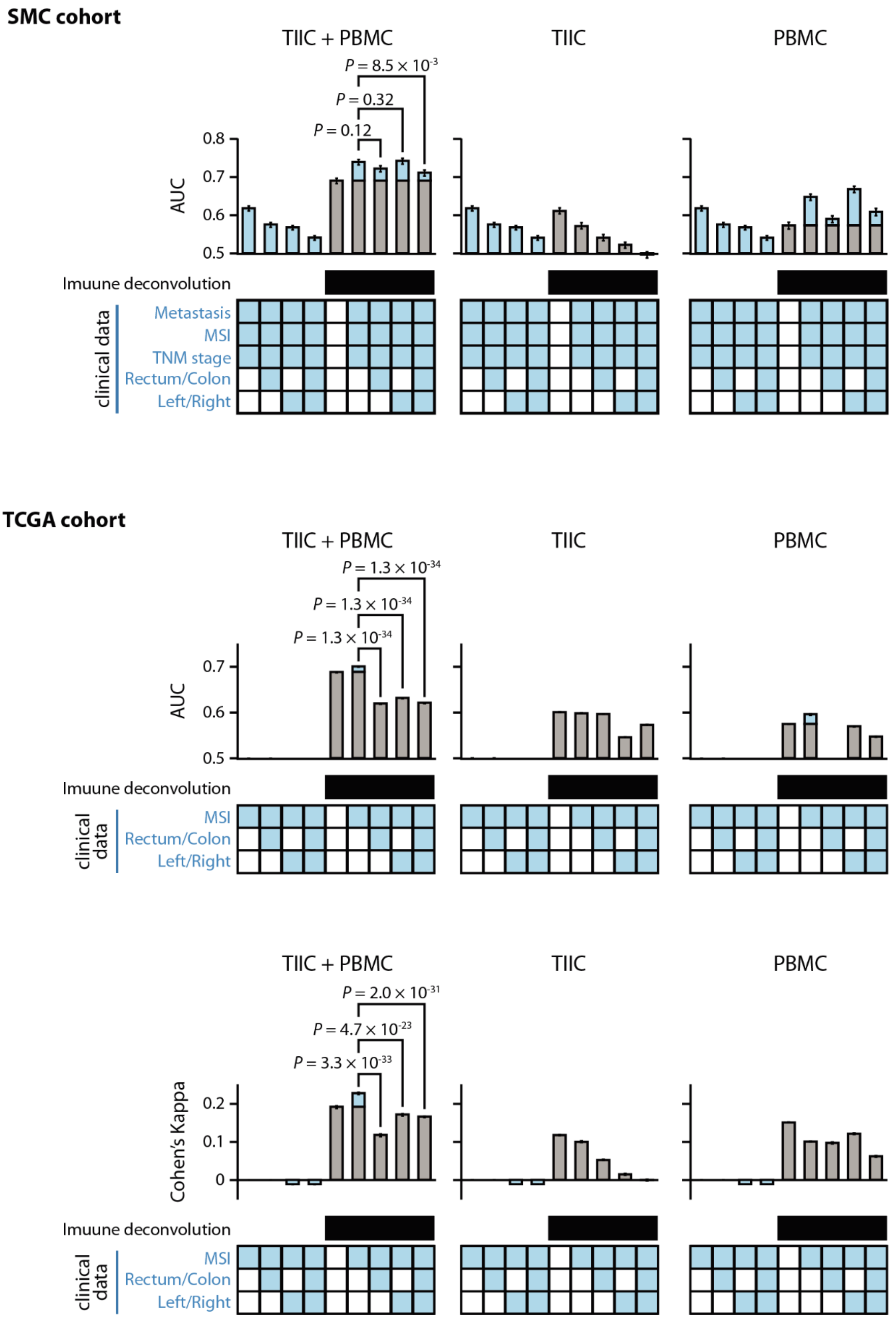

Figure S11. Performances of predicting recurrence of colorectal cancer patients from SMC and TCGA cohorts using immune cell deconvolution and tumor locations (Rectum/Colon and Left/Right). Ascending colon, hepatic flexure colon, transverse colon, and cecum were assigned as right-sided. Rectum, sigmoid colon, splenic flexure colon, st, rectosigmoid junction rectum, and descending colon were assigned as left-sided. Light

blue areas of bar graphs indicate the performance improvements when the clinical data were combined in predicting recurrence. Filled boxes are the features used in predictions. Mann-Whitney U test was performed to measure significance. Data are represented as mean ± SEM. Related to **Figure 2**.

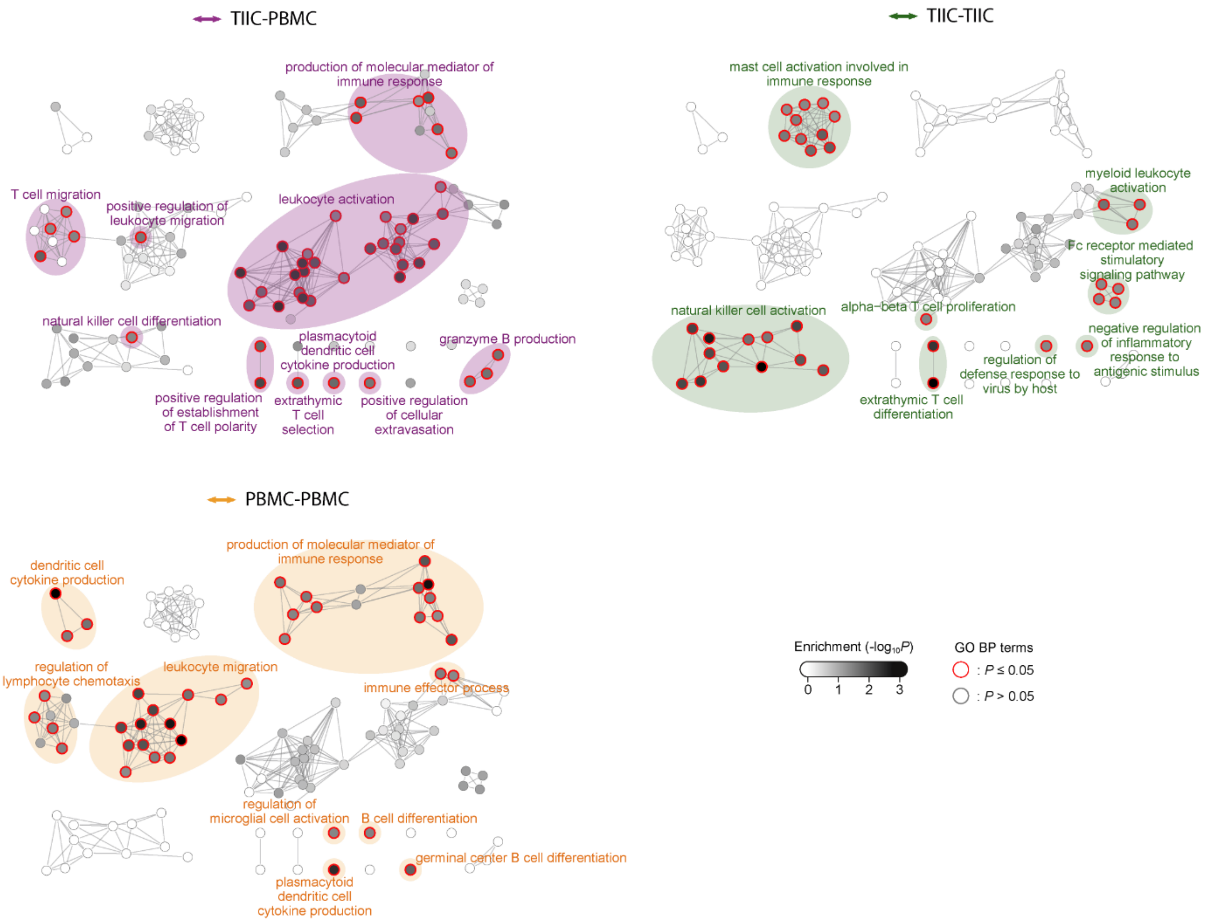

Figure S12. Functional analysis of TIIC-PBMC, TIIC-TIIC, and PBMC-PBMC DMCs in the integrative approach using GOmeth. Functional characterization of signature DMCs were performed using GOmeth, which is a method for gene ontology testing for ilumina methylation array data by normalizing the CpG density present in base-level methylation data. The nodes and edges denote the GO BP terms and their similarities. The similarities were measured by how many genes are shared using Jaccard index. Two different BP terms were linked when their Jaccard index is higher than 0.25. The color of circles indicates the significance of DMC enrichments. The clusters were labeled with the name of the GO BP term with the highest number of genes in the cluster. Related to **Figure 3**.

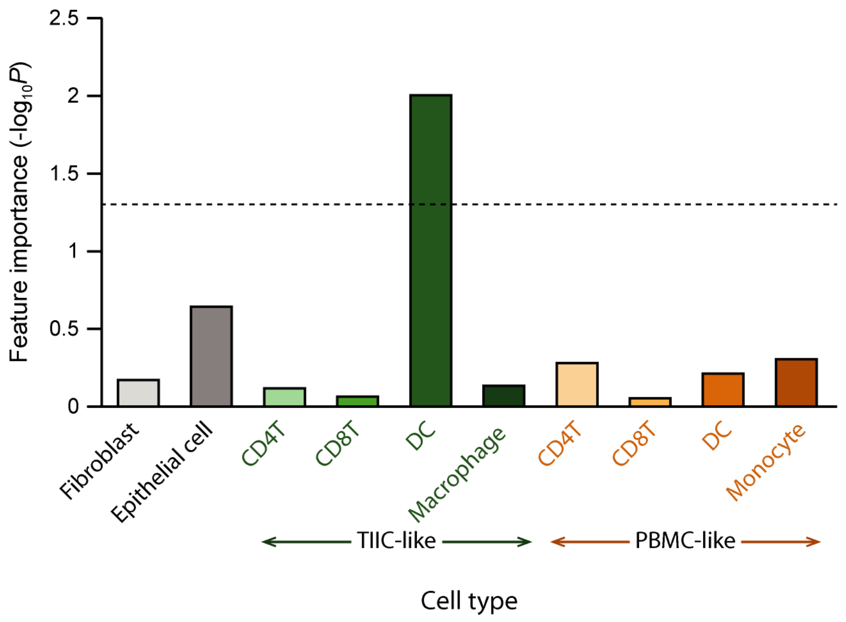

Figure S13. Feature importance of cell types to predict colorectal cancer recurrence. Feature importance was estimated using pRF, which estimates the significance of feature importance by permuting the response variable. Dotted line indicates the significance threshold (*P* value = 0.05). Related to **Figure 4**.
